# Book Reviews

**Published:** 1906-11

**Authors:** 


### BOOK REVIEWS.

THE second Report of the Welcome Research Laboratories at the Gordon Memorial College, Kartoum, under the direction of Andrew Balfour, M.D., shows that much interesting work is being done in a field that offers bright prospects for original work along many lines.

The Sudan is the territory within which these investigations are being carried on, and an excellent photograph of a dust storm gives an idea of some of the disadvantages as regards scientific work in a country where heat, dust and wind are the chief adversaries. In spite of these and many non-climatic difficulties the report makes an encouraging showing. Special analyses of the water of the Niles and their tributaries have brought out interesting and practical points of considerable importance. Sudanese gum has received the attention of Dr. Beam, who hopes to place the trade in this commodity upon a sound basis, as gum forests are at present the Sudan's chief asset.

The spread of sleeping sickness to adjacent territories lead to considerable search for the *Glossina palpalis* in the Sudan, but they are glad to make a negative report.

Dr. Neave has made many examinations of the blood of fish and birds and has found several varieties of new trypanosomes and other blood parasites.

The following from the introduction gives an idea of the wide scope of the work to be done: "The Southern Sudan is a country seamed by water-ways, on the banks of which are clustered native villages wherein all manner of rare and interesting pathological conditions are to be found. Flies and mosquitoes abound, the birds, reptiles and fish harbor strange parasites, men die from curious diseases. There is a vast field for the study of Tropical Medicine."

PROGRESSIVE MEDICINE. A Quarterly Digest of Advances, Discoveries, and Improvements in the Medical and Surgical Sciences. Edited by Hobart Amory Hare, M.D., Professor of Therapeutics and Materia Medica, Jefferson Medical College, assisted by H. R. M. Landis, M.D. Volume 3, September 1906. Lea Bros. and Company, Philadelphia and New York, Publishers.

The present volume of Progressive Medicine, like the former ones, includes all the latest advances in the various subjects discussed. The diseases of the thorax and its viscera, including the heart, lungs and blood-vessels, are thoroughly covered by William Ewart, M.D., F.R.C.P.

William S. Gottheil, M.D., takes up Dermatology and Syphilis, to which he devotes eighty-two pages. The spirochæta pallida, or treponema pallida, is reviewed and a very good micro-photograph given. He recommends most highly hypodermic medication with the salicylate of mercury in the treatment of syphilis and describes the technique of making the injections.

Nearly one hundred pages are devoted to Obstetrics by Richard C. Norris, M.D.

This volume is concluded by William G. Spiller, M.D., upon the Nervous System. He urges that gumma is not the most common lesion of cerebral syphilis.

Much valuable and interesting information is furnished to the medical profession by these various authorities in an easily assimilated form.

---

A MANUAL OF OTOLOGY. By Gorham Bacon, A.B., M.D., Professor of Otology in the College of Physicians and Surgeons, Columbia University, New York; Aural Surgeon, New York Eye and Ear Infirmary. With an introductory chapter by Clarence John Blake, M.D., Professor of Otology in Harvard University. Fourth edition, revised and enlarged. Handsome 12mo volume of 485 pages, with 134 illustrations and 11 plates. Price, cloth, $2.25 net. Lea Brothers & Co., Publishers, New York and Philadelphia, 1906.

In the rapid exhaustion of three revisions of this work the profession at large, as well as teachers of Otology have come to recognize and appreciate it as "The Standard Manual" of the subject on which it treats.

The author in this new edition has improved and added to the illustrations and has found it necessary to increase the number of pages to enable him to include all the important advances made in Otology since the publication of the third edition.

Among the new topics considered may be mentioned Osteomyelitis, Primary Jugular Bulb Thrombosis and Suppurative Inflammation of the Labyrinth, while the portions treating on Leucocytosis, Lumbar Puncture and the Treatment of Facial Paralysis, have been entirely rewritten.

---

CHEMISTRY: GENERAL, MEDICAL AND PHARMACEUTICAL, INCLUDING THE CHEMISTRY OF THE U. S. PHARMACOPŒIA. A Manual of the Science of Chemistry and its Application to Medicine and Pharmacy. By John Attfield, F.R.S., M.A., Ph.D., F.C.S., etc., Professor of Practical Chemistry to the Pharmaceutical Society of Great Britain, etc. New (19th) edition, specially revised by the author to accord with the New U. S. Pharmacopœia, edited by Leonard Dobbin, Ph.D., F.I.C., etc., Lecturer on Chemistry in the University of Edinburgh, etc. 12mo. 760 pages, illustrated. Price, cloth, $2.50, net. Lea Brothers & Co., Philadelphia and New York, 1906.

The revision of Attfield's Chemistry was confided by the author to Mr. Leonard Dobbin, of the University of Edinburgh an examiner in chemistry on the board of examiners for Scotland of the Pharmaceutical Society of Great Britain.

The work has been brought thoroughly up to date and to accord with the most recent Pharmacopœias of the United States and of Great Britain. The original plan of the book has been strictly adhered to, as seventeen large editions in the United States and eighteen in Great Britain are an evidence of the approval of the professions of medicine and pharmacy in the two great English-speaking nations of the world.

The author's ideal has been to produce a Manual of Chemistry for medical and pharmaceutical students, one in which not only the science of chemistry is taught, but in which the chemistry of every substance having interest for the followers of medicine and pharmacy, at more or less length, and in proportion to its importance and its position in relation to the leading principles of chemistry, is set forth with all attainable exactness.

---

KIEPE'S MATERIA MEDICA AND THERAPEUTICS. A Manual for Students and Physicians attending Postgraduate courses. By Edward J. Kiepe, Professor of Materia Medica in the Department of Pharmacy, and Adjunct-Professor of Materia Medica and Pharmacology in the Medical Department, University of Buffalo. In one 12mo volume of 265 pages. Cloth, $1.00, net. Lea Brothers & Co., Publishers, Philadelphia and New York, 1906.

The *Medical Epitome Series* when complete will consist of twenty-three volumes—this is the twentieth, leaving but three more to complete the set. It is easy for students, and practitioners as well, to post themselves to date for examinations or practical purposes, or attending post-graduate courses, by reading these authoritative little books. They are written by professors and teachers in colleges of high standing, and the subjects are treated in a manner as clear, thorough and interesting as the necessary limits of space will permit.

---

CIRCUMCISION.

John Knott (*Medical Record*, June 9, 1906) says that among the utilitarian motives which may have promoted the adoption of circumcision, those connected with the cleanliness and hygiene of the person seem to be very important. Again, the reflex irritation caused by the condition in children gives rise to verious disturbances. Among these are dysuria, enuresis, hernia, hydrocele, vesical calculus, prolapsus recti, epileptiform convulsions, and reflex paralysis. The writer believes that the sacrificial theory of the origin of the practice of circumcision—national and tribal—or rather the combination of the sacrificial and initiatory ceremony, would appear to furnish the most acceptable account of this widely-spread practice. In all ages and among all tribes and nations who have practiced circumcision, this operation has usually been the nucleus of some special ceremonial. The writer takes up in considerable detail the history of circumcision.

